# Blood Cell-Derived Microvesicles in Hematological Diseases and beyond

**DOI:** 10.3390/biom12060803

**Published:** 2022-06-08

**Authors:** Hara T. Georgatzakou, Sotirios P. Fortis, Effie G. Papageorgiou, Marianna H. Antonelou, Anastasios G. Kriebardis

**Affiliations:** 1 Laboratory of Reliability and Quality Control in Laboratory Hematology (HemQcR), Department of Biomedical Sciences, School of Health & Caring Sciences, University of West Attica (UniWA), 12243 Egaleo, Greece; cgeorgatz@uniwa.gr (H.T.G.); sfortis@uniwa.gr (S.P.F.); efipapag@uniwa.gr (E.G.P.); 2 Department of Biology, Section of Cell Biology and Biophysics, National & Kapodistrian University of Athens (NKUA), 15784 Athens, Greece

**Keywords:** blood, extracellular vesicles, microvesicles, microparticles, medium/large vesicles, hematological disorder, disease biomarker

## Abstract

Microvesicles or ectosomes represent a major type of extracellular vesicles that are formed by outward budding of the plasma membrane. Typically, they are bigger than exosomes but smaller than apoptotic vesicles, although they may overlap with both in size and content. Their release by cells is a means to dispose redundant, damaged, or dangerous material; to repair membrane lesions; and, primarily, to mediate intercellular communication. By participating in these vital activities, microvesicles may impact a wide array of cell processes and, consequently, changes in their concentration or components have been associated with several pathologies. Of note, microvesicles released by leukocytes, red blood cells, and platelets, which constitute the vast majority of plasma microvesicles, change under a plethora of diseases affecting not only the hematological, but also the nervous, cardiovascular, and urinary systems, among others. In fact, there is evidence that microvesicles released by blood cells are significant contributors towards pathophysiological states, having inflammatory and/or coagulation and/or immunomodulatory arms, by either promoting or inhibiting the relative disease phenotypes. Consequently, even though microvesicles are typically considered to have adverse links with disease prognosis, progression, or outcomes, not infrequently, they exert protective roles in the affected cells. Based on these functional relations, microvesicles might represent promising disease biomarkers with diagnostic, monitoring, and therapeutic applications, equally to the more thoroughly studied exosomes. In the current review, we provide a summary of the features of microvesicles released by blood cells and their potential implication in hematological and non-hematological diseases.

## 1. Introduction

Extracellular vesicles (EVs) are nanoscale membranous particles that are released by cells to the extracellular space during cell aging or following stimulation, activation, or apoptosis. They mediate (i) developmental programs, as in the case of reticulocyte maturation to red blood cells (RBC), (ii) disposal of cellular waste and potentially harmful signaling molecules, (iii) membrane remodeling or repair of damaged patches to avoid cell death, and (iv) transmission of packaged functional biomolecules and signals between cells, tissues and organisms at a distance [1]. According to their biogenesis pathway, size, and other biophysical and biochemical features, they have been traditionally categorized into three predominant classes: exosomes, microvesicles (or microparticles, or ectosomes), and apoptotic bodies.

Exosomes, the smallest among EVs (<150 nm), represent the former intraluminal vesicles of multivesicular bodies that instead of fusing with lysosomes for degradation, they fuse with the plasma membrane [2]. Their content and features reflect both their origin from the endosomal compartment, and, as it holds for all EV types, the stimulus behind their production, that determines to an extent their functions. Exosomes release, usually in a homeostatic way, by all cells that possess a functional endocytic machinery, and might be either endosomal sorting complex required for transport (ESCRT)-dependent or independent, based on local changes in the lateral organization of the endosome membrane, driven by ceramides or members of the tetraspanin family.

Microvesicles (MVs), on the other hand, are ectosomes since they are generated by ectocytosis, namely by direct outward budding of the plasma membrane. This functionally significant budding of cell surface is usually driven by Ca^2+^ related changes in the organization of the plasma membrane and cortical cytoskeleton, including lateral heterogeneity in sub-domains and transversal asymmetry of lipids, mainly phosphatidylserine (PS) and phosphatidylethanolamine (PE) [3]. Typically, they are larger (in the range of 50–1000 nm). They contain cytoplasmic and membrane components which, as for the case of exosomes, are sorted for release.

Apoptotic bodies or apoptotic vesicles, on the other hand, include the largest among EVs (50–5000 nm) that are released not by healthy or living cells but by dying via apoptosis cells and may be enriched in organelle-associated biomolecules and organelles, as well as nucleus fragments and components [4,5].

For the last two decades, EV identification has been based on properties of individual EV subgroups, like size and density, and on the presence of differential marker proteins and lipids, such as tetraspanins CD63, CD81, and CD9 for exosomes, and exposed PS for MVs [6]. More recently, however, it has become apparent that there is a great deal of overlapping in size, density, composition, z-potential, and even in biogenesis mechanisms between the various classes of EVs, which renders technically challenging the differentiation between them, and, more importantly, raises questions about in vivo functions and effects that have been ascribed to them. Moreover, it was revealed that previously established protein or lipid biomarkers of EV classes cannot be universally applied to different cell types or conditions [7].

Actually, there are small MVs in the “typical” size range of exosomes (40–100 nm) that bud directly from the plasma membrane mediated by the ARRDC1 and TSG101 proteins [8,9]. Moreover, several traditional exosome protein markers (like the CD9 or CD81) have been verified as true MV components [10,11]. To continue, it was found that apart from exosomes, the biogenesis of MVs might be ESCRT-dependent or independent, as well. Additionally, research on ciliary EVs that mediate peptidergic signaling revealed that they are topologically related to MVs since they are formed by outward budding of the ciliary membrane, though the matrix and the membrane of cilia differ substantially in lipid and protein composition compared to the cell body. Even though cilia are not present in cells of the lymphoid or myeloid lineages in mammals, ciliary EVs may be components of the biological fluids [12].

Finally, the presence of non-membranous small extracellular particles [6] and lipidic exomers [13] further complicate the analyses of EVs by increasing the heterogeneity of extracellular space in nanoparticles that may share EV features, as shown by the presence of suggested exosome markers in the exomers [14]. By using combinations of elegant and state of the art imaging and biochemical methodological approaches, reassessment of EV composition and features has been achieved in the last years and new candidate EV markers emerge potentially able to distinguish EV classes according to their biogenesis pathway (exosomes or ectosomes) irrespective of their partially overlapping size [6].

Indeed, in HeLa cells for example small MVs can be distinguished from exosomes based on a small set of surface proteins (e.g., BSG and SLC3A2 vs. LAMP1/2 and CD63, respectively) as revealed through monitoring of protein trafficking in atypical secretion pathways under variable endosomal pH, and quantitative proteomics analysis. It was demonstrated that small EVs with CD9 and CD81 (but little CD63) are MVs, while small EVs with late endosomal proteins and CD63 (but little CD9) may qualify as exosomes [15]. These results were confirmed by quantitative omics, Transmission Electron Microscopy (TEM), and Nanoparticle Tracking Analysis (NTA) approaches in plasma EVs that further propose actinin-4 and syntenin-1 as potential MV or exosome markers, respectively [7,11]. Most importantly, syntenin-1 was found consistently abundant in exosomes of different cell origin, species, and biological fluids [7]. Updating of EV composition in EVs derived by cancer lines and normal human kidney epithelial cells as well as in EVs isolated by human plasma further includes (i) annexin A1 as a novel, specific marker for MVs; (ii) absence of glycolytic enzymes, cytoskeletal elements, miRNA biogenesis, and processing machinery and externalized PS in exosomes; and (iii) lack of DNA from all small EVs [6]. With regards to EVs collected from human plasma, multiomics analyses in combination with TEM and NTA methods revealed a wider array of proteoforms in MVs compared to exosomes, a range of differentially expressed proteins and metabolites, which are related to important biological processes, and estimated size ranges to 50–150 nm vs. 100–400 nm respectively [11].

According to the updated guidelines of the International Society for Extracellular Vesicles (ISEV) [16], in the current absence of established, specific biomarkers to distinguish between exosomes and MVs, the specific subgroups of EVs should be characterized by functional terms corresponding to their physical characteristics (e.g., size), specific composition (e.g., CD81^+^), and cell origin (e.g., platelet-derived). Moreover, EV characterization should be accomplished by combinations of methods involving phenotyping, size distribution and imaging, e.g., electron microscopy, to verify at first place the vesicular nature of the analyzed material, namely, the presence of limiting lipid bilayer and membrane proteins. In cases where the identity of EV cannot be confirmed, then other terminology, e.g., extracellular particles (instead of EVs) is highly recommended.

Release of EVs by blood cells has been extensively studied, but mainly with respect to exosomes (approximately threefold higher number of articles compared to those of MVs), in the light of their dominant roles in antigen presentation and intercellular transfer of RNA molecules in many diseases. The recently suggested effects of reticulocyte-derived exosomes on several genetic non-malignant hematologic diseases is an illustrative example [17,18]. However, a role for the blood cell-derived MVs is also expected and is continuously demonstrated in several hematological diseases, including leukemias and infections for white blood cell-derived MVs (WbcMVs), thrombotic or hemorrhagic diseases for platelet-derived MVs (PMVs), and anemias for RBC-derived MVs (RMVs) [18,19,20,21,22,23,24]. In addition, increasingly more studies focus on blood cell MVs’ contribution in the pathophysiology and clinical phenotypes of non-hematological diseases affecting the nervous, endocrine, cardiovascular, and urinary systems, among others [22,25,26,27,28,29,30].

Many different methodologies have been followed for the isolation of blood cell-derived MVs by plasma, including ultracentrifugation, density gradient centrifugation, size exclusion chromatography, ultrafiltration, asymmetrical-flow field-flow fractionation, microfluidics, affinity based capturing, etc.; however, differential centrifugation is most often the technique of choice [31,32]. The protocols include initial dilution of viscous plasma, followed by preliminary centrifugations at 300× *g* and 2000× *g* to remove living and dead cells, and then centrifugation of the cell-free supernatant at 10,000–25,000× *g* to pellet MVs of the typical size, or up to 37,000× *g* to pellet smaller MVs known to derived by RBC and other blood cells. It is expected that the final supernatant is enriched in small EVs, including exosomes, that may be pelleted at forces > 100,000× *g*. Of note, several preanalytical or analytical factors (e.g., rotor type and centrifugal force) may affect the properties of the collected material.

Characterization of those MVs has been achieved either in isolated pellets or in cell-free plasma by (i) Dynamic light scattering, Nanoparticle Tracking Analysis and similar techniques to determine EV size and size distribution based on the Brownian motion of those particles; (ii) Flow Cytometry (FC) for enumeration and phenotyping of larger EVs; (iii) immunoblotting and proteomics for protein composition; (iv) metabolomics for detection of metabolites; and (v) Electron Microscopy and Atomic Force Microscopy for imaging.

The strict guidelines of the ISEV for the nomenclature and characterization of EVs and no-vesicular particles have begun to apply in the research field of blood cell derived MVs [33,34]. However, a large area in the relative literature is not in compliance with the recently reported guidelines. The majority of those works have been mostly or exclusively based on differential centrifugation for isolation and/or flow cytometry for MV characterization, despite the fact that the plasma MVs are too small to be accurately detected by FC. The availability of FC in many hematological and biological laboratories has supported numerous well designed, paired studies (e.g., patient vs. control) that have provided a valuable source of in vitro and in vivo data for MVs in health and several pathologies (Table 1, Table 2 and Table 3) [35]. Instead of ignoring this source, it can be critically assessed in the light of the widely appreciated inherent methodological limitations, keeping in mind that even the simpler techniques can separate larger EVs enriched in MVs from small EVs enriched in exosomes, and associate their properties with clinical metrics and phenotypes. It is encouraging that the reliability and accuracy of the results gradually increase in the field by new studies that verify the identity of bona fide EVs and apply appropriate combinations of methods for their characterization.

For these reasons, this review focuses on the far less appreciated (compared to the exosomes) MVs, namely, small to medium size ectosomes, derived by blood cells, their emerging relationship with several hematological and non-hematological diseases, and their potential utilization as disease biomarkers and therapeutic vectors.

## 2. White Blood Cell-Derived Vesicles

### 2.1. General Characteristics

Microvesicles are produced by all types of leukocytes, namely, neutrophils [113,114], monocytes [115], and lymphocytes [53,116,117], as identified by the presence of leukocyte-specific surface markers CD11a^+^ or CD45^+^. WbcMVs express cytoplasmic and membrane markers of parent cells, including proteins, bioactive lipids, and nucleic acids. Through pro-coagulant surface proteins and lipids they may affect blood hemostasis [118]. They also interact with innate and adaptive immunity systems by transmitting pro-inflammatory or anti-inflammatory factors, as shown in both non-infectious diseases [119] and in infections [19]. Their accumulation in blood as a result of cell stimulation and the expression of disease-specific markers, render them potential disease monitoring or prognostic factors in Amyotrophic Lateral Sclerosis [25], Acute Respiratory Distress Syndrome [26], atherosclerosis [27], Acute Myeloid Leukemia [120], and septic shock [28,121]. More specifically, in Amyotrophic Lateral Sclerosis, WbcMVs accumulation is correlated with slower disease progression, probably due to the removal of the toxic misfolded superoxide dismutase protein. In a similar way, in Acute Respiratory Distress Syndrome, a protective role has also been suggested for WbcMVs but the mechanism is still unknown. In contrast, the positive association between WbcMVs generation and plaque formation in atherosclerosis, as well as their sharp elevation in septic shock, suggest their utility as possible disease biomarkers. Finally, they also seem to have a role on endothelium homeostasis or dysfunction related to breathing disorders and to the increased cardiovascular risk associated with the obstructive sleep apnea (OSA) and pre-eclampsia [122,123,124,125] (Table 1).

### 2.2. Neutrophil-Derived MVs

Neutrophils are the most abundant granulocyte subpopulation of circulating white blood cells. Both resting and activated neutrophil granulocytes release CD66b^+^ MVs through their plasma membrane [126]. According to the status of parent cells and their microenvironment, the cell activation stimulus or stressful condition and the MV phenotype, neutrophil-derived MVs (NMVs) may exert pro-coagulant and pro- or anti- inflammatory effects. For instance, in vitro studies revealed that EVs released by resting neutrophils and annexin-A1-loaded NMVs exert anti-inflammatory actions [127,128], whereas NMVs released by opsonization-activated neutrophils promote inflammatory processes in neutrophils and endothelial cells (ECs) in vitro [128].

A significant interaction of blood cells with NMVs has been noticed in vitro. NMVs exposing phosphatidylserine (PS) [36] on their outer membrane leaflet have been shown to activate the classic pathway of complement [129]. Complement-opsonized NMVs bind to red blood cells via the complement receptor-1 [36], a process that possibly triggers their phagocytosis. On the other hand, the exposure of resting platelets (PLTs) to NMVs expressing active CD11b/CD18 integrin molecule, triggers platelet activation and expression of P-selectin in a pro-thrombotic way of effect [37].

Further immunomodulatory roles have been attributed to NMVs produced under hyperglycemic conditions [38] based on their enrichment in pro-inflammatory cytokine IL-1β^+^, a key regulator in many acute and chronic inflammatory diseases, and in sepsis, where their ingestion by phagocytes enhances their phagocytic activity and, thus, their response to the infection [20]. NMVs are also able to induce cytokine release (IL-6), TF and adhesion molecules expression in ECs in vitro [39], provoking stress signaling pathways, such as NF-kB activation, through their microRNAs enriched content (e.g., miR-155) [40]. Based on this mechanism, in vivo studies in mice have shown their implication in atherogenesis [41]. Moreover, detrimental effects of NMVs on endothelium have been observed in vasculitis, sepsis [42] and in infectious diseases, manifested through increase in blood brain barrier permeability [130,131].

Regarding their effects in the epithelia, the enrichment of NMVs in myeloperoxidase an enzyme essential for the oxidative burst activity of neutrophils impairs the proliferation and migration of intestinal epithelial cells after tissue injury, inhibiting wound healing [132].

As previously referred, the linkage of blood cell derived MVs with the pathogenesis of many diseases has suggested their use as disease biomarkers. In the case of NMVs, their accumulation in blood has been strongly associated with disease severity in sepsis [28,43,44], in non-small cell lung cancer [45,46,47], and in familial hypercholesterolemia, in which they have been suggested as biomarkers of atherosclerotic plaque burden in asymptomatic patients [48]. Moreover, their levels can be used as an independent biomarker for the prediction of mortality in infectious endocarditis [51].

Despite their negative effects, a protective role has been proposed for NMVs in rheumatoid arthritis, by mitigating the activation of macrophages [50] and inducing the release of anti-inflammatory cytokines from macrophages [49] and chondrocytes [133], partly due to the Annexin A1 expression. Additionally, granulocyte-derived MVs contain urokinase plasminogen activator receptor (uPAR) and, when loaded with urokinase (uPA), they are not only capable of lysing clots in vitro, but also reducing microthrombi in kidneys and lungs of septic mice, increasing their survival rate [134].

### 2.3. Lymphocyte-Derived MVs

Lymphocytes are peripheral blood mononuclear cells (PBMCs) that represent the 25 to 40% of circulating white blood cells. The three fundamental subpopulations of these immune cells are T-cells, B-cells, and natural killer (NK) cells. Apart from the leukocyte common antigen CD45, the lymphocyte derived MVs (LMVs) express protein markers indicative of their cellular origin, such as CD3, CD4, CD19, CD20, and CD52 (also found in monocytes and dendritic cells) and CD8 (also found in dendritic cells) [135].

Pathologically high LMV levels are encountered in hematological disturbances of the leukemia spectrum. In those cases, the majority of MVs comes from progenitor cells, either T- or B-cells expressing abnormal phenotypic characteristics. In vitro studies have proved the direct effect of LMVs originating from lymphoblastic cells on human bronchial epithelial cells [136], since their phagocytosis leads to enhanced cytokine expression and activation of apoptotic signaling, partly attributed to their caspase 3, 8, and 9-enriched content. In chronic lymphocytic leukemia, increased levels of CD52^+^ LMVs (CD52 is the most prevalent marker of peripheral lymphocytes) have been proposed as useful biomarkers for monitoring disease progression [57], and LMVs derived from B-cells for the prediction of treatment time and overall survival [137].

LMVs have seemed to play a dual role in inflammatory conditions, by promoting the production of either pro-inflammatory (namely TNF, IL-1β) or anti-inflammatory (e.g., sIL-1Ra) cytokines by human monocytes in vitro, under the regulatory effect of high-density lipoproteins (HDLs) [138]. Their levels correlate well with clinical aspects of the inflammatory autoimmune polymyositis/dermatomyositis [52], and they are further found in the lipid-rich atherosclerotic plaques of familial hypercholesterolemia patients [53].

A range of ambiguous effects have been also proposed for LMVs on vascular homeostasis and neovascularization. There is evidence that LMVs may impair EC function [139] and, for this reason, they are commonly found in increased numbers in cases of vascular reactivity, including pre-eclampsia [55] and cardiovascular diseases (CVD) [56]. On the other hand, other studies have demonstrated that they can contribute to the repair of endothelial damages by promoting NO release from ECs [140]. In a similar way, while LMVs bearing membrane-anchored development regulators (like the morphogen sonic hedgehog) seem to promote angiogenesis in mice [54,141], other studies reported anti-proliferative effects of LMVs on ECs both in vitro and in vivo, probably by inducing reactive oxygen species (ROS) generation and regulating the VEGF signaling pathways [142].

A special reactivity towards the endothelium under inflammatory conditions has been attributed to LMVs that are derived by apoptotic lymphocytes (aLMVs). In particular, Fas ligand (Fas-L) positive aLMVs can induce vascular dysfunction in vitro through activation of the membrane cell death pathway (Fas/Fas-L) and upregulation of the nuclear factor kappa B (NFkB), nitric oxide (NO) synthase and cyclooxygenase-2 proteins [59]. Their increased levels in HIV-1 infected patients have been associated with regulatory effects on dendritic cells, and, thus, with immune responses [58]. According to other studies, the aLMVs may weaken the endothelial function by affecting the production of ROS and the expression of NO synthase and caveolin-1 proteins in ECs [60]. The potential action of aLMVs in the endothelium was further highlighted by in vivo studies in diabetic patients characterized by vascular hyporeactivity [59] and in ischemic retinopathy. Additionally, a recent study highlighted the ability of aLMVs to inhibit retina angiogenesis [62]. Finally, the vast majority of the TF-related procoagulant activity of the human atherosclerotic plaques has been attributed to the presence of highly procoagulant PS^+^ TF^+^ aLMVs [61].

The accelerated release of LMVs in HIV infection, preeclampsia [55,143], type 2 diabetes mellitus, and in atherosclerosis [61] often exhibits correlations with inflammation and endothelial defects. Moreover, it has seemed to predict the clinical outcome in patients with severe bacterial infection (i.e., sepsis) and tissue damage (i.e., trauma), namely, situations characterized by inflammatory reactions and organ dysfunctions too [28].

Finally, the natural killer (NK) cells are cytotoxic lymphocytes that participate in the immunosurveillance and defense against infections and tumorigenesis/metastasis. Apart from the typical MV markers, the natural-killer-derived MVs (NKMVs) carry surface molecules and internal loads (e.g., perforin, granzymes, granulysin, CD40L, and miRNAs associated with anti-tumor activity) that can effectively support defective immunological activities, similar to those of the parent cells in vitro and in vivo, through crosstalk with both immune and infected/tumor cells [63,64]. Reports on the result of this communication refer to the activation of PBMCs (both lymphocytes and monocytes) [144], to the inhibition of proliferation and/or induction of apoptosis in several tumor cell lines, and to the migratory activity and activation of ECs following stimulation of the parent cells by IL-1beta [145]. NKMVs are encountered as a promising arrow in scientists’ quiver for cancer immunotherapy due to their immunomodulatory effects and their ability to transport antitumor drugs [146].

### 2.4. Monocyte-Derived MVs

Monocytes are PBMCs that represent 2–8% of the total number of leukocytes in the blood. They participate in the interconnections of innate with the adaptive immunity systems and of infection/inflammation with thrombosis [147].

Monocyte-derived MVs (MoMVs), namely CD14^+^ MVs, are able to promote blood clotting via expression of the procoagulant proteins TF (receptor for factor FVII/VIIa) and thrombomodulin (essential membrane cofactor of thrombin), as well as of anionic phospholipids, such as PS on their surface [147,148]. In fact, according to several studies, the second largest population of thrombogenic MVs (TF^+^) after the PLT-derived ones, is the MoMVs [66,149,150]. In addition, MoMVs released by cells exposed to activated protein C (a natural anticoagulant protein) in vitro express the endothelial protein C receptor [151] and activated protein C [151], that has factor V and factor VIII inactivating activity. Moreover, despite the fact that MoMVs may bear TF [152], the TF activity can be markedly inhibited by the co-expression of TF pathway inhibitor on the same vesicles. However, TF expression may induce accumulation of platelets at the site of vascular damage and, thus, indirectly trigger thrombosis. Along with TF, P-selectin glycoprotein ligand-1 expression has also been observed on MoMVs [153]. The interaction of MoMVs with ECs and activated platelets through the vesicular P-selectin ligand at the thrombus site is associated with TF accumulation, thrombin generation, and rapid blood clotting, as shown by both in vitro and in vivo studies [118,153,154]. Consequently, the relative ratio of TF and TF pathway inhibitor expression in MoMVs tilts the scales of their activity towards either thrombosis or hemorrhage [155].

There is concrete evidence of the significant role of MoMVs in endothelium disorders observed in inflammatory diseases and/or hypercoagulable states [66]. The lipopolysaccharide (LPS)-induced activation of monocytes results in TF-expression and increased thrombomodulin activity, as well as release of membrane vesicles that exhibit TF, prothrombinase and thrombomodulin activity [147]. However, the production of IL-10 by the LPS-treated cells has inhibitory effects on TF expression on both cells and MVs [150]. At the same time, the MoMVs IL-1 can induce inflammation-driven activation of ECs [156]. In sickle cell anemia (a hematological disease characterized by hemolytic, inflammatory, and vaso-occlusive aspects) the activation of monocytes leads to TF expression and release of procoagulant TF^+^ MoMVs, the levels of which further increase under sickle crisis in close correlation with other typical coagulation markers [67]. Additionally, elevated levels of MoMVs have been observed in systemic inflammatory diseases, such as chronic kidney disease [157], pre-eclampsia [65,68], and Chronic Obstructive Pulmonary Disease (COPD) [69], either as a result of the inflammatory state and monocyte activation (in pre-eclampsia and COPD) or as contributors to it (in uremia).

The TF^+^ MoMVs released by stimulated THP-1 monocytes in vitro have negative effects on EC (including membrane blebbing, increased TF-related cell surface thrombogenicity, PS exposure, and apoptosis), but they may promote angiogenesis [66]. Further apoptotic effects of MoMVs carrying senescence markers have been reported in vascular smooth muscle cells exposed to exogenous inflammatory stimuli (e.g., uremia, endotoxins, HIV-1 infection etc.) followed by vascular dysfunction [19,115,158]. The high concentration of MoMVs and PLT-derived MVs in patients with diabetes and hypertension [70,159] are considered risk factors for cardiovascular complications and mortality [48,71,73] and biomarkers of disease progression [72]. Similar associations have been reported in type 2 diabetes [160], diabetic retinopathy [72], COPD [69], acute myocardial infarction severity, and CVD. In most cases, those MoMV effects seemed to be exerted via their TF and mitochondrial cytochrome oxidase activities, the latter reflecting monocytes’ response to hypoxic conditions [73].

In conclusion, WbcMVs may be actively implicated in vascular homeostasis, through the regulation of EC function, and in blood clotting by both pro- or anti-coagulant effects. These effects can be combined with immunomodulation, mainly through induction of cytokines’ release by other cells. Further interesting activities of WbcMVs that have been confirmed in vitro include neovascularization, as well as anti-tumor and anti-proliferative ones.

### 2.5. White Blood Cells Microvesiculation in Storage Conditions

Even though WbcMVs characteristics have not been extensively studied under storage conditions (sWbcMVs), neither of their effects after transfusion, a time course increase in WbcMVs (along with other blood cell types-derived MV) levels has been observed, especially in non-leukoreduced RBC units during storage. Neutrophil activation has been also found correlated to PLT vesiculation whereas activation of PLTs and their interaction with leukocytes during storage, induces leukocytes apoptosis and death, a process exacerbating their vesiculation rate [161]. All these data, along with the previously mentioned effects of aLMVs on deregulation of vascular homeostasis support a potential contribution of sWbcMVs to post-transfusion adverse effects. Simultaneously, a recent study by Pinheiro et al. [162] reported that the majority of cytokines released under storage conditions is encapsulated in TGFβ^+^ MVs (probably coming from T regulatory lymphocytes), supporting the immune-modulatory effects of donor sWbcMVs after transfusion, a characteristic further confirmed by in vivo studies in mice.

## 3. Red Blood Cell Vesiculation

RBC vesiculation occurs throughout their life in the circulation, though it is accelerated in normal aging and under oxidative, metabolic, and mechanical stresses or eryptosis. Many studies have reported that the size of RMV in plasma is rather small, ~150 nm. Since the red blood cells are devoid of any endosomal network, and though they release nanovesicles in the size range of exosomes both in vitro and in vivo [163], these nanovesicles are released (in a mandatory way) by outward budding of the plasma membrane. The RMVs are positive for the erythroid specific surface marker CD235^+^ (glycophorine A), they are enriched in Hb and modified Hb species, contain band 3, redox enzymes and GPI-proteins, and expose PS. They release as a result of oxidative stress and/or Ca^2+^ accumulation leading to activation of Ca^2+^-dependent enzymes and PS externalization, commonly seen in aging, osmotic imbalances, exposure to xenobiotics, and eryptosis. The role of RMVs under homeostasis conditions has not been fully identified. However, their presence in high numbers in the circulation has been associated with a decelerated thrombin formation indicating an anticoagulant role [164]. This role is partly attributed to their capacity for binding the anticoagulant protein S [165]. On the other hand, there is evidence that the RMVs can act as a compensatory factor to both primary and secondary hemostasis defects, namely platelet dysfunction or coagulation factors disorders, respectively [166]. In vivo and in vitro studies have proved that this procoagulant capacity may be exerted in a FVIIa-, TF-, and PS-dependent manner [167,168,169]. Finally, the hemoglobin containing RMVs can also affect NO bioavailability, potentially leading to impairment of endothelium-dependent vasodilation [170,171].

### 3.1. RBC Vesiculation in Disease

Membrane vesiculation of RBCs is increased in many hematological diseases including hereditary hemolytic anemias, myelodysplastic or myeloproliferative syndromes, and paroxysmal nocturnal hemoglobinuria (PNH), as well as in non-hematological diseases like atherosclerosis and CVD, Parkinson disease, diabetes (type 2), OSA, inflammatory disorders, and Malaria parasitemia, among others (Table 2).

The RBCs show an accelerated rate of vesiculation in hematological diseases, such as hemolytic anemias, myelodysplastic syndromes, and PNH [21,22,29]. Oxidative stress and complement activation seem to be the main promoting factors, as reflected in their enrichment in lipid and protein oxidation markers, including oxidized hemoglobin, catalase, and peroxiredoxin-2, namely, proteins involved in the redox homeostasis [21,172,173]. Another factor, the increased levels of plasma thrombospondin-1 seem to trigger the RMV shedding in SCD, as observed by in vivo studies [75]. Pathologically increased levels of PS^+^ RMVs have been considered a pro-thrombotic phenotype in these pathologies [22,74,174,175]. In complement-mediated hemolytic disorders, the RMVs are supposed to stimulate thrombin generation in a FVIII- and FIX-dependent manner, which (apart from PS externalization) is tightly linked to the presence of iron ions [176]. Another mechanism by which RMVs contribute to thrombotic events, vasodilation/vasoconstriction and endothelial activation in PNH and other diseases is related with the regulation of the bioavailability of NO in the plasma [177,178]. RMVs are further supposed to have a pro-oxidant effect on ECs in myeloproliferative neoplasms (MPNs) and they seemed able to induce expression of adhesion molecules and release of cytokines by ECs in sickle cell disease (SCD), contributing to occlusive events and endothelial damage [178]. On the other hand, the increased levels of PS exposure on RMVs may affect their own lifespan in the circulation, suggesting a different effect in thrombogenesis, as it has been proposed for PNH [76]. Regarding their role in coagulation homeostasis, it is clearly reflected in bleeding disorders such as certain myelodysplastic syndromes, which are characterized by decreased RMVs levels [23]. Regarding their utility as disease severity biomarkers, it has been shown that the RMV levels have positive correlations with clinical markers of SCD (namely occlusive events and aortic stiffness) [30,75] as well as with the enzymatic activity of G6PD in G6PD deficient patients [77].

Several RBC membrane fragility diseases are associated with increased release of RMVs. In hereditary spherocytosis, the membrane vesiculation leads to anemia due to the preferential destruction of non-deformable and highly vesiculated spherocytes in the spleen [179]. On the same hand, RBC membrane modifications in SCD among other enhance SMase activation leading to generation of sphingosine and sphingosine 1-phosphate and MV release. Treatment with amitryptiline, a functional inhibitor of a-SMase, reduces MV generation both in vitro and in vivo. As suggested by the authors, this mechanism could be applicable to other RBC disorders [180].

The augmented formation of RMVs has been also supposed to be a contributing factor to the endothelial dysfunction observed in many non-hematological pathological states. For example, the sharp increase in RMVs concentration observed in myocardial infarction [80,81] exhibits positive correlations with clinical markers of endothelial injury [80]. Additionally, intermittent hypoxia in patients with OSA provokes RMV release through ROS and iCa^2+^ stresses [78]. It has been reported by in vitro studies that the detrimental effect of RMVs on eNOS activity is a causal factor for the observed endothelial dysfunction and hypertension in OSA patients [78,79]. In cellular microenvironments enriched in cytokines (as in the case of several inflammatory conditions), the RMVs are supposed to offer a substrate for the production of bioactive lipids (such as arachidonic acid and lysophosphatidic acid) through phosphatidylserine exposure, that have a negative impact on endothelial function and vascular integrity [181]. Additionally, in the malaria infection, engulfment of RMVs by macrophages or infected RBCs induces changes in their inflammatory phenotype and in the developmental stage of the parasite, respectively [82]. Finally, enrichment of RMVs from Parkinson disease patients in the toxic protein a-synuclein constitutes them as potential biomarkers of disease progression and classification [86,87,182].

In contrast to the above mentioned evidence for a negative involvement of RMVs in the clinical phenotypes and/or pathophysiology of some diseases, in animal models of ischemic preconditioning, which is a protective response of heart against damages imposed by incoming ischemic episodes, the plasma RMVs have shown to exert cardioprotective effects through ameliorating the endoplasmic reticulum stress-specific apoptosis of cardiomyocytes, attributed in part to the down-regulation in the expression of endoplasmic reticulum molecular chaperone GRP78, transcription factor CHOP, and caspase 12 [85]. Moreover, there is evidence that the release of RMVs constitutes a mechanism of innate resistance of RBCs to malaria infection, at least in vitro, based on the finding that the transfer of some vesicular miRNA complexes to the parasites downregulates the expression of critical malaria antigens [183]. Not least at all, the artificial production of micro- and nano-RMVs for use in a wide array of biomedical applications constitutes an active area of research on advanced therapeutic strategies that are based on drug delivery systems [84,184]. A good example can be taken from the field of malaria, in which EVs derived from Plasmodium falciparum-infected RBCs exhibit higher internalization rate compared to EVs derived by non-infected RBCs. The delivery of hydrophobic antimalarial drugs through those EVs, result in better therapeutic outcome in comparison to that reached following supplementation of free drugs [183].

Conclusively, the RBCs respond to the systemic oxidative stress, complement activation and dilute stress factors present in the plasma under several disease contexts by releasing MVs. Subsequently, the RMVs communicate through their surface and cargo molecules with cellular (e.g., macrophages) and dilute (e.g., coagulation factors) interactors to induce or support thrombotic or immunomodulatory reactions leading to endothelial damage/activation and changes in plasma homeostasis. However, the RMVs may also have protective effects, as reported in ischemic preconditioning and in malaria, while both native and induced RMVs are being intensively studied as drug delivery systems.

### 3.2. Red Blood Cell Microvesiculation in Storage Conditions

Studied as a separate group of MVs, due to the completely different cellular and plasma environment, low temperature, and absence of clearance mechanisms, microvesicles derived from RBCs stored under blood bank conditions (sRMVs) also show some interesting features [185]. First of all, they are accumulated exponentially in the supernatant of the RBC units by the storage time and their composition seems to follow the in vitro aging of the parent cells, whereas these characteristics are also affected by the specific storage conditions, for example the storage medium [83,186]. Moreover, they are characterized by increased fibrinolytic activity due to the presence of plasminogen on their surface (that decreases, however, by the storage time), as well as to antithrombin activity, probably related with the presence of a2-macroglobulin [187,188]. On the other hand, the sRMVs (particularly the PS^+^ ones) might be highly procoagulant. In fact, they are able to activate two different pathways that end up to FIX activation, predisposing probably to the inflammatory and thrombotic complications of transfusion therapy [189,190]. Transfusion Related Immune Modulation (TRIM) might be also associated with sRMVs through interactions with cells that change the cytokine secretome profile or the survival dynamics of peripheral blood mononuclear cells [191]. The immunomodulatory effects of sRMVs are actually confirmed by the production of pro-inflammatory cytokines by bone marrow-derived macrophages in vitro, probably constituting a risk factor for transfusion related- adverse effects [192]. sRMVs accumulation in the presence of neutrophils triggers ROS production, suggesting another mechanism towards the induction or promotion of transfusion-related acute lung injury (TRALI) [193]. At the same time, the vesicular hemoglobin is almost as effective as the cell-free hemoglobin in NO-scavenging, impairing the endothelial-dependent vasodilation [171]. In other cases, however, like the severe PNH, transfusion of RBC units enriched in RMVs is considered beneficial due to the transfer of proteins that protect against the complement attack [194].

## 4. Platelet Vesiculation

There is evidence that the majority of plasma MVs is derived from platelets (PLTs) [35], mainly as the result of calcium stress-induced cell activation. The hemostatic dynamics of PMVs has been mainly attributed to PS exposure, though their surface TF can also initiate the extrinsic coagulation pathway [6]. On the other hand, binding of the natural anticoagulant protein S on PMVs may ascribe anticoagulant properties to them, as happens with the RMVs [195]. The PMVs may also be active in immunomodulation, with either pro-inflammatory [196] or anti-inflammatory effects [197,198]. Some platelet surface markers are CD41, CD61, CD42a, and PAC1.

### 4.1. PLT Vesiculation in Disease

In hematological disorders characterized by a bleeding predisposition, such as the Scott syndrome and certain myelodysplastic syndromes, PLTs show a reduced vesiculation rate [23,24,89]. Conversely, it is considered that the high PMV levels in children with immune thrombocytopenic purpura restrain the severe bleeding [90]. In the same context, their accumulation in thrombocytopenia and myeloproliferative neoplasms (despite the low PLT levels) has been linked to hypercoagulable state and thrombotic events [91,92,93,94], whereas in thrombocythemia constitutes a predisposing factor for venous thrombosis [95,96,97] (Table 3). All the aforementioned characteristics highlight their important regulatory role in hemostasis.

In many settings, the PMVs are considered links in the chain of events that promote the atherosclerotic lesion, namely, inflammation-hypercoagulability-neovascularization [199]. Along with the RMVs, the increased levels of PMVs [98] have been suggested as a biomarker of SCD severity, in terms of either vaso-occlusive events [98], or acute lung injury, that is proceeded through an IL-1-dependent pathway [99]. Actually, even though the majority of plasma MVs is mainly derived from platelets even in physiological [35,200,201] or in pathological states, as stated above [86,104], in SCD and in Parkinson disease, this ratio is disturbed by the predominance of RMVs [67,86]. PMVs accumulation has been further suggested as a disease severity biomarker or a predisposing factor for adverse effects in CVD, metabolic syndrome, and OSA. More specifically, PMVs levels have been proposed as risk factors for atherosclerosis in CVD [100], and early coagulation markers in type II diabetes [101,102]. They have been also associated with increased risk for CVD and disease severity biomarkers in OSA [103,104,175], with thrombotic events in end stage renal disease [105,106], and with both glycemia and oxidative stress markers in Metabolic Syndrome patients [88] (Table 3). Their presence in the joints of arthritic patients and in models of autoimmune inflammatory arthritis in vivo [107], as well as the fact that they may gain egress to lymphatic vessels through joint vascular leakage, actually confirm their implication in RA pathogenesis [108]. Finally, as opposed to the previously mentioned pro-thrombotic effects of the abundant PS^+^ PMVs in ESRD [105,106], some researchers suggest that PS^+^ PMVs exhibit reduced procoagulant activity in end stage renal disease patients compared to healthy controls [109].

However, a protective, anti-inflammatory role of PMVs to the endothelium has also been shown in sepsis, based on the finding that specific microRNA cargo can restrain the activation of ECs in vitro [110,202]. In the same context, increased levels of PMVs have been associated with better disease outcomes with respect to mortality, disseminated intravascular coagulation (DIC) and organ dysfunction [111,121]. Accordingly, in vitro studies have shown a significant contribution of PMVs after ischemia-reperfusion preconditioning towards a post-ischemic blood vessel formation [112] and endothelial damage restoration through the stimulation of early outgrowth cells to produce angiogenic growth factors [203]. Finally, examination of engineered, drug-loaded PMVs, both in cancer cell lines and in leukemia patients, highlighted their ability to support advanced and targeted therapeutic schemes characterized by low off-target toxicity and high biocompatibility [204].

To sum up, the main role of platelets as coordinators of coagulation and hemostasis processes is reflected in the PMVs. Under pathological settings, high PMV levels enhance the risk for atherosclerotic events and CVD and promote inflammation. Their anti-inflammatory effects in sepsis and restorative roles to damaged vessels after ischemia-reperfusion preconditioning highlights a heterogeneous, multifaceted functionality, the biological parameters of which deserve further studying.

### 4.2. Platelet Microvesiculation under PLT Storage Conditions

There is a limited number of studies on PMVs isolated from PLT units stored in blood bank conditions (sPMVs). These studies suggest their involvement in FXII- and FXI-dependent thrombin generation, mainly through the surface exposure of PS [205,206].

## 5. Conclusions

MVs are released from all blood cells, either constitutively or in response to various stimuli, including oxidative stress, aging, apoptosis, and intake of signals by other cells. MV roles include discarding of damaged, useless, or potentially dangerous biomolecules, membrane repair, and transfer of functional biomolecules and bioactive signals and messages to target cells or cellular microenvironments. The rate of EV release is affected by several local or systemic pathological settings, where oxidative, mechanical, and metabolic stresses, as well as inflammation, prevail. In these cases, MVs constitute a cellular response to stress, a defensive mechanism to restore membrane and cell homeostasis and ensure survival, and an effective way for the dissemination of any single local event to the biological system. By sharing the typical behavior of biological vesicles, the MVs mainly serve communication purposes, the effects of which might be as complex as their heterogeneity.

**Figure 1:**
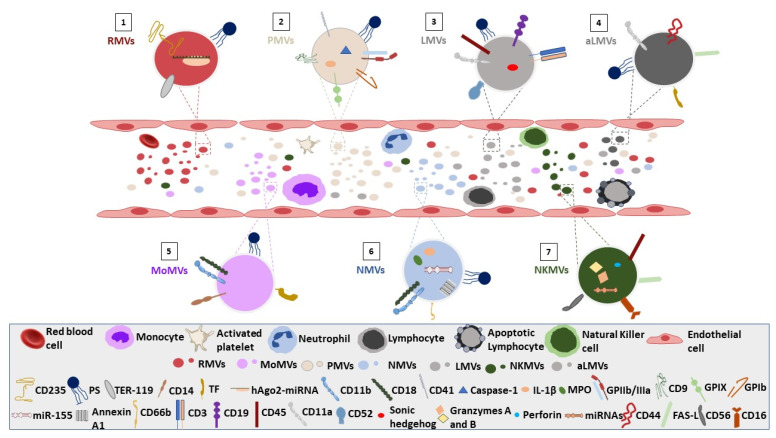
Immunophenotyping characteristics of blood cell-derived microvesicles. (1) RMVs characterized by the cell-specific marker CD235. PS acts as an “eat-me” signal and prothrombotic marker. The presence of human argonaute 1-micro ribonucleic acid antigen in MVs has been shown to be associated with innate resistance of RBCs to malaria infection, whereas TER-119 antigen has a cardioprotective effect. (2) PMVs are recognized by the platelet-specific markers, CD41 and CD61. They exert their hemostatic dynamics through their PS exposure and TF expression which can initiate the extrinsic coagulation pathway. Interleukin 1 beta and caspase-1 presence in PMVs can induce vasoocclusion is sickle cell disease whereas GPIb, GPIIb/IIIa, GPIX, and CD9 have been associated with calcification on acute coronary syndrome and constitute independent predictors for thrombotic and atherothrombotic events. (3) CD3, CD19, and CD11a levels on LMVs show strong correlation with inflammatory diseases (e.g., inflammatory autoimmune polymyositis/dermatomyositis) and are accumulated in the lipid-rich atherosclerotic plaques of familial hypercholesterolaemia patients. A range of ambiguous effects have been also proposed for LMVs on vascular homeostasis (CD11a) and neovascularization (sonic hedgehog protein). (4) aLMVs express CD44, Fas-L, CD11a, PS, and TF playing an important role on dendritic cells function, endothelial function impairment, vascular hyporeactivity induction, and in blood hemostasis. (5) Monocyte-derived MVs (MoMVs) have procoagulant activity via expression of the procoagulant proteins (TF and thrombomodulin) and PS on their surface. Moreover, MoMVs expressing CD14, CD18, and PS impact on endothelial cell dysfunction or damage (6) Neutrophil-derived microvesicles (NMVs) exposing PS on their outer membrane leaflet have been shown to activate the classic pathway of complement--. NMVs expressing active CD11b/CD18 integrin molecule, trigger platelet activation. miR-155 enriched NMVs induce cytokine release and MPO positive NMVs promote endothelial cells damage and vascular dysfunction. (7) Natural-killer-derived MVs (NKMVs) carry internal and surface molecules (e.g., perforin, granzymes, granulysin, CD40L, and miRNAs associated with anti-tumor activity) that can inhibit proliferation and induce apoptosis of tumor cell lines. **RMVs**, Red blood cell-derived microvesicles; **MoMVs,** Monocyte-derived microvesicles; **PMVs**, Platelet-derived microvesicles; **NMVs**, Neutrophil-derived microvesicles; **LMVs**, Lymphocyte-derived microvesicles; **NKMVs**, Natural Killer-derived microvesicles; **aLMVs**, Apoptotic Lymphocyte-derived microvesicles; **CD235**, glycophorin-A, **PS**; phosphatidylserine; **TER-119**, TER-119 antigen; **CD14**, lipopolysaccharide receptor; **TF**, Tissue factor; **hAgo2-miRNA**, Human argonaute 1- micro Ribonucleic acid (RNA); **CD11b**, Macrophage-1 antigen; **CD18**, Integrin beta chain-2; **CD41**, Integrin alpha chain 2b; **IL-1β**, Interleukin 1 beta; **GPIIb/IIIa**, Glycoprotein IIb/IIIa; **CD9**, a member of the transmembrane 4 superfamily; **GPIX**, Glycoprotein IX; **GPIb**, Glycoprotein Ib; **miR-155**, microRNA-155; **CD66b**, Glycosylphosphatidylinisotol (GPI)-anchored, highly glycosylated protein belonging to the carcinoembryonic Ag supergene family; **MPO**, Myeloperoxidase; **CD3**, T-cell surface antigen; **CD19**, B-lymphocyte antigen; **CD45**, Leukocyte common antigen; **CD11a**, integrin alpha L chain; **CD52**, CAMPATH-1 antigen; **miRNA**, microRNA; **CD44**, Homing cell adhesion molecule (HCAM); **CD56**, Neural cell adhesion molecule; **CD16**, FcγRIII.

Their abundance (or shortage, more rarely) in the bloodstream has been increasingly associated with the pathogenesis, progression, complications, and dissemination (and thus, with the response to therapies) of hematological and non-hematological diseases, that have immunological, coagulation, and/or inflammatory arms. Most often, variation in MV features is associated with damaged, activated, or dysfunctional endothelia, dysregulation of hemostasis, and immunomodulation. More rarely, it is associated with apoptosis, or resistance to it, neovascularization, and wound healing. In both cases, the emerging networks of blood cell-derived MV effects reveal hidden aspects of disease heterogeneity and a more systemic way by which certain disease phenotypes are shaped, namely, by EV-based transfer of biological response modifiers (Figure 1).

Until today, the basic issues that researchers face up in studying MVs have not been fully resolved. Widely applied isolation techniques do not ensure purity. Widely applied enumeration techniques are not appropriate for the smaller MVs. High-resolution techniques are not appropriate for enumeration, or analysis of large number of MVs. The (not usually available) “single particle” nanotechnologies may not perform high resolution imaging [16]. However, as MVs emerge as diagnostic tools in a similar way as exosomes do, a deeper understanding of MV biogenesis, contents, targeting, and intake/interaction mechanisms is highly needed. The ISEV’2018 recommendations for adopting a new way of EV nomenclature and appropriate combinations of techniques to verify the presence of bona fide EVs in the biological preparations and characterize them, as well as usage of the recently introduced reliable MV-specific probes and elegant methodological approaches, including live cell imaging, spatial proteomics, and imaging flow cytometry seem to be one-way road to better understanding of their biology, clinical effects, and medical applications, provided that the gained knowledge will be effectively translated into clinically friendly laboratory protocols for their assessment. So far, the collected evidence supports the notion that a great deal of understanding of EV biology is tightly interconnected to that of cell membrane biology.

## Figures and Tables

**Table 1:** White blood cell- derived microvesicles’ source, detection methods, immunophenotypic characteristics, function, and association with diseases progression/initiation/prognosis or potential utility as disease biomarkers.

White Blood Cell-Derived MVs							
MVs Type	MVs Source	Isolation/Characterization Method	MVs Phenotype	Function/Role	Disease	Effects/Relation to Disease	References
Neutrophil-derived MVs (NMVs)	Stimulation of isolated PMNs (cell cultures)	Centrifugation/Flow Cytometry	PS^+^ NMVs	Classic pathway of complement activation	Systemic inflammation diseases		[36]
	Stimulation of isolated PMNs (cell cultures)	Centrifugation/Flow Cytometry	CD11b^+^ CD18^+^ MVs	Platelet activation	Atherosclerosis, Chronic Prothrombotic States, Cardiovascular Diseases	Pro-thrombotic effects	[37]
	Stimulation of isolated PMNs (cell cultures)	Centrifugation/Flow Cytometry	IL-1β^+^ NMVs	Immunomodulatory roles	Hyperglycemia		[38]
	Stimulation of isolated PMNs (cell cultures)	Centrifugation/Flow Cytometry	miR-155 enriched NMVs	Induce cytokine release by ECs and stress signaling pathways	Vascular Inflammation, Atherosclerosis	Endothelial dysfunction, Atherosclerotic plaque development	[39,40,41]
	Stimulation of isolated PMNs (cell cultures)	Differential centrifugation/Flow Cytometry	MPO^+^ NMVs	ECs damage and vascular dysfunction	Vasculitis, Sepsis		[42]
	Blood plasma	Differential centrifugation/Flow Cytometry	CD66b^+^ MVs accumulation	Pro-inflammatory effects	Sepsis, Trauma, Non-Small Cell Lung Cancer	Clinical outcome, Disease progression	[28,43,44,45,46,47]
	Blood plasma	Differential centrifugation/Flow Cytometry	CD11b^+^ CD66^+^ PS^+^ MVs accumulation		Familial Hypercholesterolemia	Cardiovascular risk and coronary calcification and atherosclerotic plaque burden biomarker	[48]
	Stimulation of isolated PMNs (cell cultures)	Centrifugation/Flow cytometry	Annexin A1^+^ NMVs	Anti-inflammatory cytokines release	Rheumatoid Arthritis	Protective role	[49,50]
	Blood plasma	Differential centrifugation/Flow Cytometry	CD66b^+^ MVs accumulation		Infectious Endocarditis	Independent predictor of mortality	[51]
Lymphocyte-derived MVs (LMVs)	Blood plasma	Differential centrifugation/Flow Cytometry & TEM	CD3^+^ and CD19^+^ MVs accumulation	Pro-inflammatory role	Polymyositis/Dermatomyositis (PM/DM)	Possible role in the pathogenesis of PM/DM	[52]
	Blood plasma	Differential centrifugation/Flow Cytometry	CD45^+^ CD3^+^ PS^+^ MVs accumulation		Familial Hypercholesterolaemia Patients	Markers of lipid-rich atherosclerotic plaques	[53]
	Human lymphoid CEM T cell line (cell cultures)	Differential centrifugation/Immunoblot analysis	Sonic hedgehog^+^ LMVs	Endothelial damage repair, neovascularization	Ischemic Cardiovascular Diseases		[54]
	Blood plasma	Differential centrifugation/Flow Cytometry & Elisa kit	CD11a^+^ MVs accumulation	Pro-inflammatory effects on vessels, endothelial dysfunction in arteries	Pre-eclampsia		[55]
	Blood plasma	Differential centrifugation/Flow Cytometry & Elisa kit	CD45^+^ CD3^+^ PS^+^ MVs accumulation		Cardiovascular Diseases (CVD)	Potential prognostic biomarkers of incident CVD	[56]
	Primary CLL B-cells	Differential centrifugation/Flow Cytometry	CD52^+^ MVs accumulation		Chronic Lymphocytic Leukemia	Disease progression	[57]
Apoptotic Lymphocyte-derived MVs (aLMVs)	Apoptotic human lymphoid CEM T cell line (cell cultures)	Differential centrifugation/Flow Cytometry	CD44^+^ aLMVs accumulation	Inhibition of dendritic cells function	HIV-1	Promising therapeutic targets	[58]
	Apoptotic human lymphoid CEM T cell line (cell cultures) & Blood plasma	Differential centrifugation/Prothrombinase assay & Immunostaining	Fas-L^+^ aLMVs accumulation	Endothelial function impairment, vascular hyporeactivity induction	Diabetes, Inflammatory diseases		[59,60]
	Atherosclerotic plaque-derived aLMVs	Differential centrifugation/Prothrombinase assay & Elisa assay	CD11a^+^ PS^+^ and TF^+^ aLMVs	Procoagulant activity	Atherosclerosis	Plaque thrombogenicity determinants	[61]
	Apoptotic human CEM T lymphocytes line (cell cultures)	Differential centrifugation/Flow Cytometry	PS^+^ aLMVs	Retinal angiogenesis suppression	Ischemic retinopathy	Promising therapeutic approach	[62]
Natural Killer-derived MVs (NKMVs)	Stimulation of NK Cell Line	Differential centrifugation/DLS & Immunoblot analysis	Perforin, granzymes A and B, granulysin, FasL and miRNAs enriched NKMVs	Inhibit proliferation and induce apoptosis of tumor cell lines		Potentially effective, safe, and universal immunotherapeutic agents	[63,64]
	Blood samples	Differential centrifugation/Flow Cytometry	Low levels of CD45^+^ CD16^+^ CD56^+^ MVs	Defect in active NK cell death induction	Pre-eclampsia		[65]
Monocyte-derived MVs (MoMVs)	Stimulation of monocyte cell line (THP-1)	Centrifugation/Flow Cytometry	CD18^+^ CD14^+^ PS^+^ TF^+^ MVs	Induction of endothelial thrombogenicity and apoptosis	Inflammatory diseases and hypercoagulable states	Endothelial cell dysfunction	[66]
	Blood plasma	Differential centrifugation/Flow Cytometry	CD14^+^ PS^+^ TF^+^ accumulation in sickle crisis	Procoagulant activity	Sickle Cell Disease	Contribution to thrombotic occlusive events (e.g., stroke)	[67]
	Blood plasma	Differential centrifugation/Flow Cytometry	CD11b^+^ and CD14^+^ MVs accumulation		Pre-eclampsia	Probably a systemic inflammatory response marker	[68]
	Blood plasma	Differential centrifugation/Flow Cytometry	CD14^+^ MVs accumulation	Contribute to Chronic obstructive pulmonary disease exacerbations (unknown mechanism)	Chronic obstructive pulmonary disease	Potential predictive biomarker	[69]
	Blood plasma	Differential centrifugation/Flow Cytometry	CD14^+^ MVs accumulation	Cardiovascular complications	Hypertension, hyperlipidemia with type II diabetes	Potential therapeutic target	[70]
	Blood plasma	Differential centrifugation/Flow Cytometry	CD14^+^ PS^+^ MVs accumulation	Vascular endothelial damage	Type II diabetes mellitus	Potential biomarker of CVD complication	[71]
	Blood plasma	Centrifugation/Flow Cytometry	CD14^+^ PS^+^ MVs accumulation	Enhancement the procoagulant activity, Adhesion molecules activation by ECs	Diabetic Retinopathy	Microvascular occlusions development, Potential biomarker of diabetic retinopathy progression	[72]
	Blood plasma	Centrifugation and Magnetic beads/NTA, TEM, PCR	CD14^+^ MVs expressing low levels of mitochondrial cytochrome oxidase, subunit I (MT-COI)		Coronary Artery Disease	Predictive marker for CVD risk	[73]

CVD, cardiovascular disease; DLS, Dynamic Light Scattering; ECs, endothelial cells; HIV-1, Human Immunodeficiency Virus-1; IL, interleukin; MPO, myeloperoxidase; MVs, microvesicles; PCR, Polymerase Chain Reaction; PS, phopshatidyleserine; TEM, Transmission Electron Microscopy.

**Table 2:** Red blood cell-derived microvesicles’ source, detection methods, immunophenotypic characteristics, role, and association with diseases progression/initiation/prognosis and potential utility as disease biomarkers.

Red Blood Cell-Derived MVs						
MVs Source	Isolation/Characterization Method	MVs Phenotype	Function/Role	Disease	Effects/Relation to Disease	References
Blood plasma	Differential centrifugation/Flow Cytometry	CD235^+^ PS^+^ MVs accumulation	Thrombin activation, Nitric Oxide bioavailability disruption	Sickle Cell Disease and Thalassaemia Intermedia	Potential pro-thrombotic marker	[22,74]
Blood plasma	Differential centrifugation/Flow Cytometry	CD235a^+^ PS^+^ MVs accumulation		Myeloproliferative neoplasms		[29]
Blood plasma	Differential centrifugation/Flow Cytometry	CD235a^+^ MVs accumulation	Positive correlation with aortic stiffness, pulmonary artery pressure, and tricuspid regurgitant velocity	Sickle Cell Disease	Potential biomarker for vascular dysfunction and disease severity	[30]
TSP-1 stimulated RBCs, isolated from blood	Centrifugation/Flow Cytometry	CD235a^+^ PS^+^ MVs accumulation	Endothelial cell damage, vascular dysfunction, renal vaso-occlusion	Sickle Cell Disease (mice)		[75]
Blood plasma	Differential centrifugation/Flow Cytometry	CD235a^+^ PS^-^ and CD235a^+^ CD59^-^ MVs accumulation	Disturbed contribution to hemostasis and thrombosis	Paroxysmal Nocturnal Hemoglobinuria		[76]
Blood plasma	Differential centrifugation/Flow Cytometry, Atomic Force Microscopy	CD235a^+^ PS^+^ MVs accumulation	Positive correlation with G6PD enzyme activity	G6PD deficiency	Potential biomarker of G6PD deficiency severity	[77]
Stimulated RBCs, isolated from blood	Ultracentrifugation/NTA, Immunoblotting analysis and TEM	CD235a^+^ MVs accumulation	Disturbed the homeostasis of vascular tone, hypertension induction	Obstructive Sleep Apnea	Endothelial dysfunction marker, Obstructive Sleep Apnea severity	[78,79]
Blood plasma	Differential centrifugation/Flow Cytometry and TEM	CD235a^+^ PS^+^ MVs accumulation	correlation with several coronary artery diseases and adverse clinical events	Myocardial Infarction		[80,81]
RBCs infected with P. falciparum (cell cultures)	Differential centrifugation, filtration, 60% sucrose cushion/Flow cytometry, TEM and Immunoblotting analysis	RMVs-derived from Malaria Infected RBCs	Immunomodulatory properties, Transmission stage parasite development stimulation	Malaria Infection		[82]
RBCs infected with P. falciparum (cell cultures)	Differential centrifugation/Flow Cytometry, PCR, Immunoblotting analysis	Enriched in hAgo2-miRNA complexes RMVs	innate resistance of RBCs to malaria infection	Malaria Infection	Therapeutic potential as drug delivery systems	[83,84]
Blood plasma	Differential centrifugation/Flow Cytometry, TEM	TER-119^+^ MVs accumulation	Cardioprotective effects	Ischemic preconditioning		[85]
Blood plasma	Immuno-capture method/NTA, Immunoblotting analysis and TEM	Enriched in a-synuclein CD235a^+^ MVs	Rapid crossing of the blood-brain barrier	Parkinson Disease	Progression or initiation disease biomarker	[86,87]
Blood plasma	Differential centrifugation/Flow Cytometry	PS^+^ CD235a^+^ MVs accumulation	Association with hyperlipidemia	Metabolic Syndrome		[88]

RBCs, red blood cells; G6PD, Glucose-6-phosphate dehydrogenase; NTA, Nanoparticle Tracking Analysis, PCR, Polymerase Chain Reaction; PS, phosphatidyleserine; TEM, Transmission Electron Microscopy.

**Table 3:** Platelet-derived microvesicles’ source, detection methods, immunophenotypic characteristics, role, and association with diseases progression/initiation/prognosis and potential utility as disease biomarkers.

Platelet-Derived MVs						
MVs Source	Isolation/Characterization Method	MVs Phenotype	Function/Role	Disease	Effects/Relation to Disease	References
Blood plasma	Differential centrifugation/Flow Cytometry	CD41^+^ MVs accumulation	Positive correlation with aortic stiffness, pulmonary artery pressure, and tricuspid regurgitant velocity	Sickle Cell Disease	Potential biomarker for vascular dysfunction and disease severity biomarker	[30]
Blood plasma/In vitro stimulation of platelets	Differential centrifugation/NTA & Flow Cytometry	Low levels of CD41^+^ MVs	Reduced or defected procoagulant activity	Scott syndrome, myelodysplastic syndromes	High bleeding risk	[23,24,89]
Blood plasma	Differential centrifugation/Flow Cytometry	CD41^+^ MVs accumulation	Hypercoagulability state and thrombotic events, Pro-thrombotic tendency	Immune thrombocytopenic purpura, Thrombocytopenia, Myeloproliferative Neoplasms	Protection against bleeding events	[90,91,92,93]
Blood plasma	Differential centrifugation/DLS, Flow Cytometry	CD41^+^ MVs accumulation	Thrombotic events	Thrombotic Thrombocytopenic Purpura		[94]
Blood plasma	Differential centrifugation/Flow Cytometry	CD41^+^ MVs accumulation	Pro-thrombotic potential	Thrombocythemia	Risk factors for thrombosis	[95,96,97]
Blood plasma	Differential centrifugation/Flow Cytometry	CD41^+^ PS^+^ MVs accumulation in severe SCD	Vaso-occlusive events	Sickle Cell Disease	Disease severity biomarker	[98]
Blood plasma	Differential centrifugation/NTA, Elisa kit, Immunoblotting analysis	IL-1β^+^ and caspase-1^+^ PMVs accumulation	Lung vaso-occlusion	Sickle Cell Disease	Potential therapeutic targeting	[99]
Blood plasma	Differential centrifugation/Elisa kit	-GPIb^+^ GPIIb/IIIa^+^ GPIX^+^ CD9^+^ PMVs accumulation (Elisa kit)	Correlation with calcification	Acute Coronary Syndrome	Independent predictor for thrombotic events, atherothrombotic events risk	[100]
Blood plasma	Differential centrifugation/Flow Cytometry	CD42a^+^ PS^+^ MVs accumulation	Association with glycemic profile	Newly diagnosed diabetes mellitus type 2	Early markers of thrombosis	[101]
Blood plasma	Differential centrifugation/Flow Cytometry	CD41^+^ PS^+^ MVs accumulation	Correlation with glycemic markers	Diabets Mellitus type 2	Potential biomarkers for Diabets Mellitus type 2	[102]
Blood plasma	Differential centrifugation/Elisa kit	GPIb^+^ GPIIb/IIIa^+^ GPIX^+^ CD9^+^ PMVs accumulation (Elisa kit)	Correlation with apneahypopnea index	Obstructive Sleep Apnea	Potential biomarker of increased cardiovascular risk	[103]
Blood plasma	Differential centrifugation/Flow Cytometry	CD41a^+^ PS^+^ MVs accumulation	Correlation with OSA severity indicators	Obstructive Sleep Apnea	Potential OSA severity biomarker	[104]
Blood plasma	Differential centrifugation/Flow Cytometry	CD41a^+^ PS^+^ MVs accumulation	Association with thrombotic events	End Stage Renal Disease	Potential triggering factor for thrombotic events	[105]
Blood plasma	Differential centrifugation/TEM, NTA, Immunoblotting analysis	CD42b^+^ MVs accumulation	Procoagulant activity	End Stage Renal Disease	Potential mediators or predictors of occlusive cardiovascular events	[106]
Blood plasma	Differential centrifugation/Flow Cytometry	GpΙΙb/ΙΙΙa^+^ PS^+^ MVs accumulation	Correlation with glycemia and oxidative stress markers	Metabolic Syndrome	Potential marker of predisposal for diabetes	[88]
In vitro stimulation of platelets	Differential centrifugation/Flow Cytometry	Internalization of CD41^+^ MVs by neutrophils	Inflammatory phenotype of neutrophils induction	Rheumatoid Arthritis	Implication in rheumatoid arthritis pathogenesis	[107,108]
Blood plasma	Differential centrifugation/Flow Cytometry	CD41^+^ PS^+^ MVs accumulation	Low procoagulant activity	CKD stage 4		[109]
Blood plasma	Differential centrifugation/Flow Cytometry, PCR	CD41a^+^ PS^+^ MVs containing miR-223 accumulation	miR-223-mediated anti-inflammatory effects	Sepsis	Protective role against sepsis-related vascular inflammation	[110]
Blood plasma	Differential centrifugation/Flow Cytometry	Low CD41^+^ MVs levels		Disseminated Intravascular Coagulation (in sepsis)	Better disease outcome	[111]
Blood plasma	Differential centrifugation/Flow Cytometry	CD41^+^ MVs accumulation	Angiogenic activity	Chronic Ischemia		[112]

DLS, Dynamic Light Scattering; IL, interleukin; NTA, Nanoparticle Tracking Analysis, PCR, Polymerase Chain Reaction; PS, phopshatidyleserine; TEM, Transmission Electron Microscopy.
